# Deep-Eutectic-Solvent-Based Mesoporous Molecularly Imprinted Polymers for Purification of Gallic Acid from *Camellia* spp. Fruit Shells

**DOI:** 10.3390/ijms232113089

**Published:** 2022-10-28

**Authors:** Dianling Shen, Yu Yan, Xiaopeng Hu, Yujun Zhong, Zhiyang Li, Yaping Guo, Lianwu Xie, Deyi Yuan

**Affiliations:** 1 College of Sciences, Central South University of Forestry and Technology, Changsha 410004, China; 2 College of Material Science and Technology, Central South University of Forestry and Technology, Changsha 410004, China; 3 Key Laboratory of Cultivation and Protection for Non-Wood Forest Trees of Ministry of Education, Central South University of Forestry and Technology, Changsha 410004, China

**Keywords:** *Camellia* spp. fruit shells, gallic acid, molecularly imprinted polymers, deep eutectic solvents, separation and purification

## Abstract

To produce antioxidant substances from agricultural waste *Camellia* spp. fruit shells before their further utilization, gallic acid from five kinds of *Camellia* spp. fruit shells was separated on specific recognition by deep eutectic solvent molecularly imprinted polymers (DES@MIPs), which were prepared by bulk polymerization using gallic acid as the template and deep eutectic solvents (α-methylacrylic acid and choline chloride) as functional monomers. The optimized DES@MIPs were characterized by scanning electron microscopy, particle size analysis, nitrogen sorption porosimetry, elemental analysis, Fourier transform infrared spectroscopy, and thermal gravimetric analysis. The adsorptive behavior of gallic acid on DES@MIPs was also investigated. The results indicated that DES@MIPs were successfully prepared as mesoporous materials with average pore diameter of 9.65 nm and total pore volume of 0.315 cm^3^ g^−1^, and the adsorption behavior was multilayer adsorption and pseudo-second-order kinetics with the saturation adsorptive capacity of gallic acid reaching 0.7110 mmol g^−1^. Although the content of gallic acid in five fruit shells was quite different, the purification recovery of gallic acid was high, ranging from 87.85–96.75% with a purity over 80%. Thus, the purification of gallic acid from *Camellia* spp. fruit shells could be realized feasibly using DES@MIPs with favorable economic and environmental benefits.

## 1. Introduction

Gallic acid (GA) is a polyphenolic compound that is widely found in fruits and plants. Due to its anti-inflammatory, anticancer, and antioxidant properties, GA is often manufactured into antibacterial agents, nutritional supplements for cancer prevention, and as an ingredient in many cosmetics [1]. However, the precise separation of GA becomes a challenge in natural products due to complex sample matrices [2,3]. Some studies have shown that *Camellia* spp., especially *Camellia oleifera*, contain GA with high bioactivity [4,5,6]. *C. oleifera* is one of the four major woody oil crops in China, with a wide variety of species, mainly distributed in the southern region, with a planting area of 4 million hectares [7,8]. *C. oleifera* consists of fruit shells and seeds, of which the fruit shells account for 50–60% of the total weight of *C. oleifera* [9]. As agricultural waste, *C. oleifera* fruit shells are often used as fuel [10] in practical applications. Research and application on the preparation and purification of gallic acid from *Camellia* spp. fruit shells are of great significance for environmental protection as well as resource utilization of agricultural waste.

Conventional methods for extracting GA mainly include solid phase extraction [11], solvent extraction [12], ultrasonic-assisted extraction [13,14], supercritical fluid extraction [15], and other technologies. Some conventional extraction methods are time-consuming [16] and are not suitable for separation and purification of trace analogues in complex systems [2]. In contrast, the molecularly imprinted polymers (MIPs) are effective adsorbents for the separation of target substances from complex samples.

MIPs are porous polymers with specific recognition ability for target molecules or structural analogues, enabling precise separation of target substances in complex systems. They are obtained by using template molecules polymerized with functional monomers and cross-linkers. After the template molecules are removed, cavities matching the spatial configuration of the template molecules are formed to achieve highly selective recognition of target substances in complex samples [17,18].

Frequently used functional monomers are acrylic acid (AA) [19,20] and methacrylic acid (MAA) [21,22]. In contrast, MIPs prepared with deep eutectic solvents (DESs) have higher adsorption capacity and selectivity than conventional MIPs without DESs [23]. Ma et al. used DESs as functional monomers to prepare MIPs for the selective separation of catechins in black tea [24]. Li et al. reported that the DES of mixing ChCl with GA was used as both a template and functional monomer to prepare MIPs for the enrichment of GA from red ginseng tea [25,26]. In addition, a particular attraction of a DES is its tremendous structural designability, which stems in large part from the broad classes of available hydrogen bond acceptor (HBA) and hydrogen bond donor (HBD) classes [27]. With properties of low toxicity and green manufacturing, DESs would partly take the place of highly toxic functional monomers.

A DES is formed by mixing HBA such as quaternary ammonium salts or quaternary phosphorous salts with HBD such as alcohols, amides, or carboxylic acids by thermochemical treatment. Just like ionic liquids, a DES usually has a melting point close to room temperature and is green, inexpensive, easy to obtain, and easy and efficient to prepare imprinted polymers [28].

**Figure 1:**
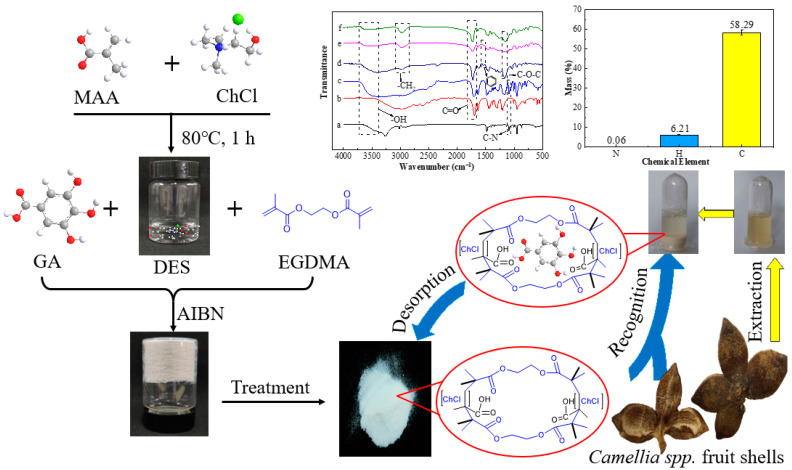
The schematic process for DES@MIPs’ preparation and characterization, and their utilization for purification of gallic acid from *Camellia* spp. fruit shells.

In this work, the specific recognition abilities of DES-based MIPs (DES@MIPs) were evaluated under different preparation conditions (the dosage of template, the molar ratio of template to cross-linker, and the type of functional monomer) with adsorptive capacity as the indicator. In addition, the adsorptive kinetics and thermodynamics of GA on DES@MIPs were also analyzed to explore the adsorptive behavior between the DES@MIPs and GA. Then, DES@MIPs were applied to the recognition of GA from five kinds of *Camellia* spp. fruit shells (Figure 1). After recognition, DES@MIPs were washed with water, eluted with eluent, and the pure GA was obtained from the dried eluate. The results showed that MIPs based on the DES have superior adsorption capacity for GA compared to previous MIPs made without the DES by the same research group [29], and it could be applied in the field of accurate separation of target substances from complex systems.

## 2. Results and Discussion

### 2.1. Influence of Polymerization Conditions on Recognition Properties of MIPs

#### 2.1.1. Functional Monomer

**Figure 2:**
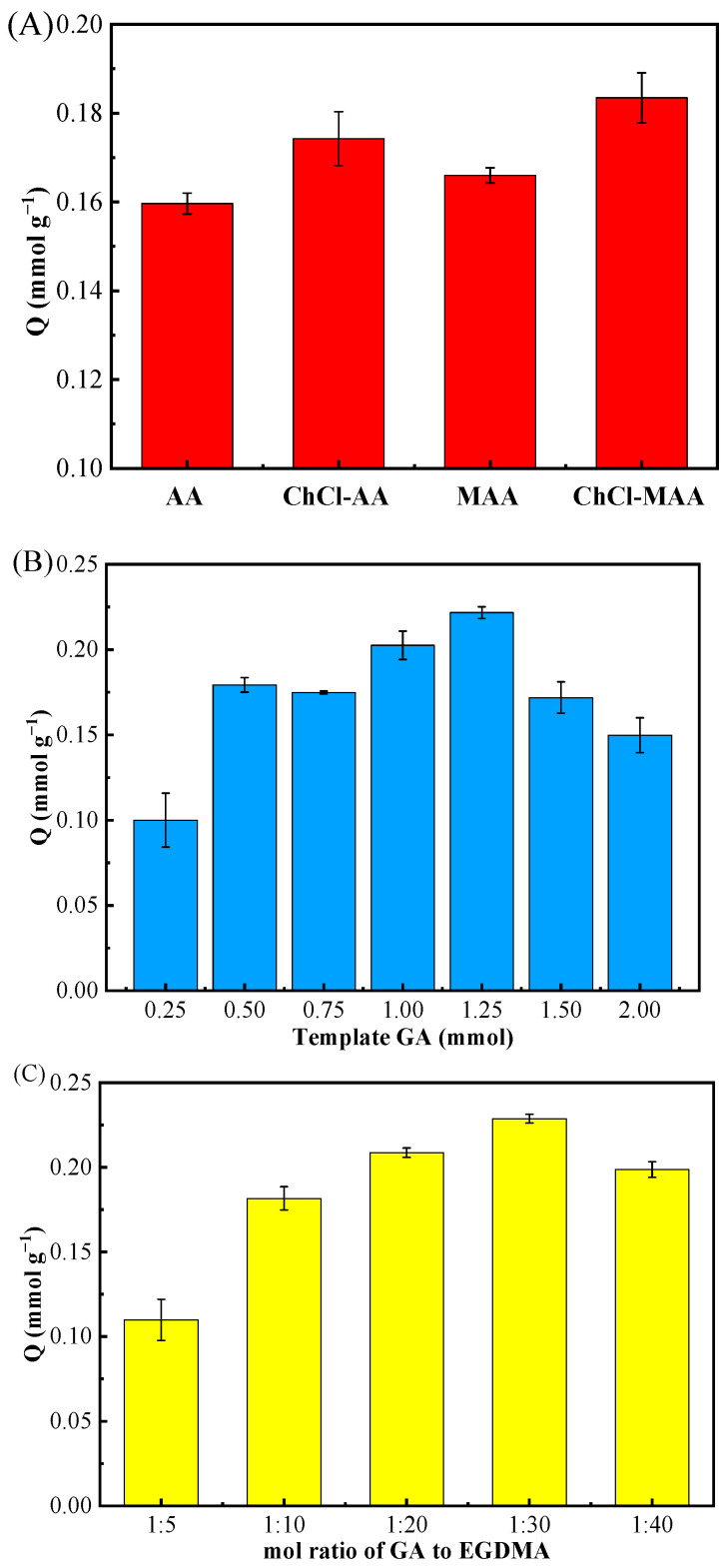
Effect of different functional monomers (AA, ChCl-AA, MAA, ChCl-MAA) (**A**), amount of template GA (0.25, 0.50, 0.75, 1.00, 1.25, 1.50, 2.00 mmol) (**B**), and molar ratio of the template GA to cross-linker EGDMA (1:5, 1:10, 1:20, 1:30, 1:40) (**C**) on MIPs’ adsorption for GA.

It is well known that the functional monomer plays an essential part in the adsorption properties of MIPs. In this work, four distinct functional monomers MAA, AA, [(ChCl-MAA)DES, and (ChCl-AA)DES] were used to prepare MIPs and NIPs for the adsorption study. The synthesis conditions were as follows: the dosage of GA was 0.5 mmol; the molar ratio of the template molecule, functional monomer, and cross-linker was 1:4:20; and AIBN/(functional monomer + cross-linker) was 1% (*w*/*w*). The adsorption results (Figure 2A) demonstrated that the adsorptive capacity of GA on MIPs reached the highest when ChCl and MAA were used as DESs.

According to the structure of GA, –OH on benzene ring can easily form a hydrogen bond with other groups possessing extra electronics since the adjacent carbonyl group has an electron-absorbing effect [30]. In addition, because GA is an organic acid, it may interact electrostatically with the Ch^+^ of ChCl [31]. Thus, the adsorptive capacity of MIPs when using (ChCl-MAA)DES and (ChCl-AA)DES as functional monomers were better than those of MAA and AA (Figure 2A). Since MAA has one more -CH_3_ group than AA, it has better polymerization ability, and the dimerization effect of MAA can enhance the molecular blotting selectivity [32]. From the results of the adsorption experiments, (ChCl-MAA)DES is strongly recommended as the functional monomer for the preparation of MIPs.

#### 2.1.2. The Dosage of Template

The dosage of the template is a key factor influencing the adsorption capacity of MIPs toward target substances [19,33]. In this study, the amount of template was researched as follows. With (ChCl-MAA)DES (ChCl:MAA = 1:2, ChCl = 1 mmol, MAA = 2 mmol) as the functional monomer, when the molar ratio of the template molecule to cross-linker was 1:20 with AIBN/(functional monomer + cross-linker) as 1% (*w*/*w*), the dosage of GA as the template in the complex material was investigated from 0.25 mmol to 2.00 mmol. The adsorption results of different MIPs prepared by different dosages of templates are shown in Figure 2B.

A low dosage of template resulted in fewer template–monomer complexes, which led to fewer binding sites in MIPs. However, when the dosage of the template was higher than 1.5 mmol, excessive template–monomer complexes produced high non-specific binding capacity, which reduced the binding selectivity [34]. Thus, the above-mentioned 1.25 mmol GA in the reaction system was best one to synthesize (ChCl-MAA)DES@MIPs.

#### 2.1.3. The Molar Ratio of the Template to Cross-Linker

For the optimal molar ratio of template to cross-linker EGDMA for the adsorption of GA, five distinct ratios (1:5, 1:10, 1:20, 1:30, 1:40) were investigated with the dosage of GA as 1.25 mmol, the functional monomer as (ChCl-MAA)DES (ChCl:MAA = 1:2, ChCl = 1 mmol, MAA = 2 mmol), and AIBN/(functional monomer + cross-linker) as 1% (*w*/*w*).

In the preparation of MIPs, the amount of cross-linker directly affects the rigidity of the polymer and plays an essential role in the stability of the rebinding site. If the amount of cross-linker is too little, the degree of cross-linker is not enough, and the synthesized MIPs cannot maintain stable cavities, leading to low recognition ability. Furthermore, high concentrations of cross-linker can make the polymer too rigid to achieve the balance between the imprinted polymer and template molecules, resulting in poor imprinting effect [30]. From the results in Figure 2C, it can be observed that the optimal molar ratio of template GA to cross-linker EGDMA was 1:30, which had great rigidity and had an excellent imprinting effect on GA.

### 2.2. Characterization of (ChCl-MAA)DES@MIPs

#### 2.2.1. Analysis of FT-IR and EA Results

**Figure 3:**
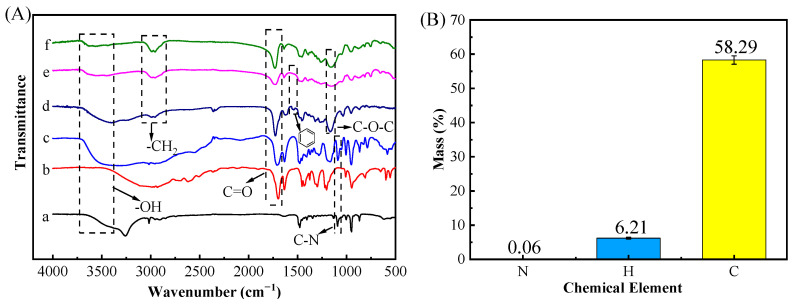
FT-IR spectra (**A**) of materials including ChCl (a), MAA (b), DES (c), (ChCl-MAA)DES@MIPs before removing template (d), (ChCl-MAA)DES@MIPs after removing template (e), (ChCl-MAA)DES@NIPs (f), and elemental analysis of (ChCl-MAA)DES@MIPs after removing template (**B**).

FT-IR (Figure 3A) was performed to ensure successful preparation of the DES and (ChCl-MAA)DES@MIPs. The structure of the DES changed after synthesis, because the DES had a strong broad peak especially at high wave numbers of 3700–3000 cm^−1^ belonging to O-H vibration, and was wider than that of ChCl, which was caused by the formation of a hydrogen bond [20]. The peak of the DES at 1091 cm^−1^ (C-N) had no significant changes, indicating that Ch^+^ had no loss [35]. The peak at 1708 cm^−1^ (C=O) in the DES was attributed to methacrylic acid group fraction [22], which also appeared in the spectra of unreacted MAA. In the FT-IR spectra of MIPs, the split double peak between 2950–3000 cm^−1^ (asymmetric and symmetric stretching of C-H) indicated a successful double bond addition of MAA in the DES [36].

Otherwise, EA data (Figure 3B) showed that MIPs contained 0.06% N, 58.29% C, and 6.21% H, indicating that MIPs contained the skeleton of ChCl. Additionally, the peak at around 1167 cm^−1^ (C-O-C) in MIPs was attributed to EGDMA, and it can be confirmed that the DES reacted with other reagents as a complete system. Comparing the MIPs before and after removing GA, the MIPs before removing the template exhibited the peak at 1533 cm^−1^ (benzene ring) from GA, but the spectra of MIPs after removing the template and NIPs did not show the same characteristic peaks, which proved that the template had been completely removed [37]. Thus, FT-IR and EA data showed that the (ChCl-MAA)DES@MIPs were successfully prepared in this study.

#### 2.2.2. Morphological Characterization by SEM

**Figure 4:**
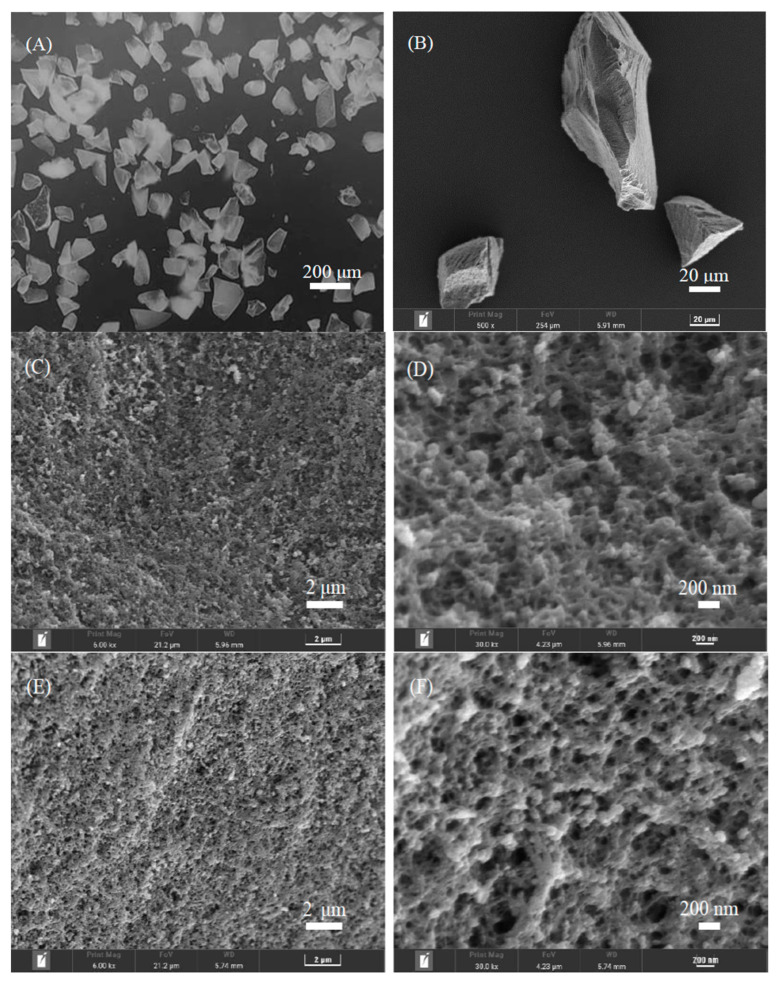
Morphology characterization of (ChCl-MAA)DES@MIPs (**A**,**B**,**C**,**D**) and (ChCl-MAA)DES@NIPs (**E**,**F**) determined by SEM.

The morphology of the crushed polymers ((ChCl-MAA)DES@MIPs and (ChCl-MAA)DES@NIPs) produced by the bulk polymerization method was assessed by SEM (Figure 4), which is useful for inspections of microstructures. The bulk polymerization process resulted in the preparation of polymer particles with a non-spherical shape (particle size of 45–75 μm), having a high degree of agglomeration and a high number of cavities. The structural characteristics revealed typical patterns on the MIPs’ and NIPs’ surfaces. The surface of (ChCl-MAA)DES@MIPs were more rough and porous, confirming the formation of GA memory sites during the process of polymerization, which facilitated the capture of the template from samples. The smoother surface of the (ChCl-MAA)DES@NIPs showed the lack of similar porous structure [38,39].

#### 2.2.3. Specific Surface Area and Particle Size

**Figure 5:**
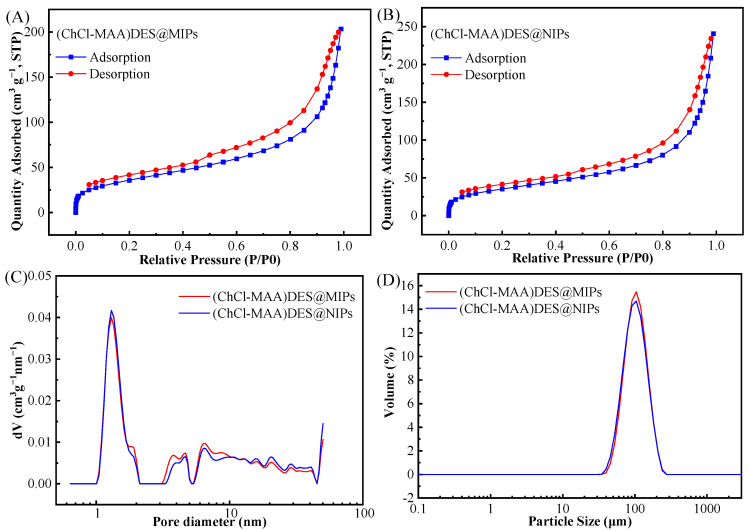
Nitrogen adsorption–desorption isotherms of (ChCl-MAA)DES@MIPs (**A**) and (ChCl-MAA)DES@NIPs (**B**), pore size distribution (**C**), and particle size distribution (**D**) of (ChCl-MAA)DES@MIPs and (ChCl-MAA)DES@NIPs.

To demonstrate that the prepared MIPs have a surface porous structure, BET-specific surface areas of (ChCl-MAA)DES@MIPs and (ChCl-MAA)DES@NIPs were measured as shown in the result of N_2_ adsorption experiments (Figure 5). According to the IUPAC nomenclature, Figure 5A,B display incomplete type IV curves of N_2_ adsorption–desorption isotherms with type H3 hysteresis loops [40]. The type of hysteresis loops indicated that the (ChCl-MAA)DES@MIPs and (ChCl-MAA)DES@NIPs were fissure hole materials. Figure 5C,D show that the particle size and pore size distribution of MIPs and NIPs were almost similar to each other. Table 1 shows the specific surface area, average pore size, and total pore volume. The average pore diameter of (ChCl-MAA)DES@MIPs and (ChCl-MAA)DES@NIPs are 9.65 nm and 11.64 nm, so they are mesoporous materials. The average pore size and total pore volume of (ChCl-MAA)DES@MIPs were smaller than those of (ChCl-MAA)DES@NIPs. However, the specific surface area of (ChCl-MAA)DES@MIPs was larger than that of (ChCl-MAA)DES@NIPs, which indicated that there are imprinted pores in (ChCl-MAA)DES@MIPs. It was the imprinted pores that increased the number of micropores and the specific surface area of MIPs.

#### 2.2.4. TGA Analysis

**Figure 6:**
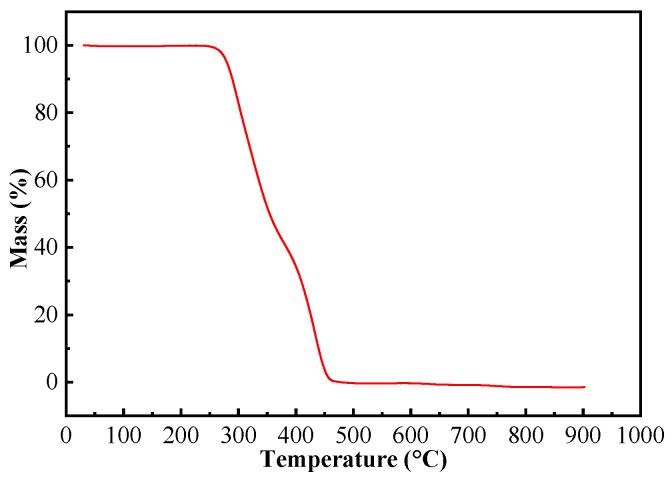
TGA curve of (ChCl-MAA)DES@MIPs.

For the application of (ChCl-MAA)DES@MIPs at room temperature, their TGA data (Figure 6) were evaluated. The weight loss of the composite was very small when the temperature was rising until 300 °C, which means that the MIPs of this work could be applied stably at room temperature [41,42].

### 2.3. Adsorption Behavior of (ChCl-MAA)DES@MIPs

For studying the kinetics and isothermal adsorption behavior of the MIPs prepared, the concentrations for a series of standard GA were detected by HPLC to obtain the standard curve. The linear regression equation (*y* = 8,702,227.55*x* + 549,911.01, where *y* is the peak area of chromatogram and *x* is GA concentration), linear range (0.25–4.00 mmol L^−1^), correlation coefficient (R^2^ = 0.9954), limit of detection (0.0674 mmol L^−1^), limit of quantitation (0.2246 mmol L^−1^), and relative standard deviation (2.37%) were obtained beforehand.

#### 2.3.1. Adsorption Kinetics

**Figure 7:**
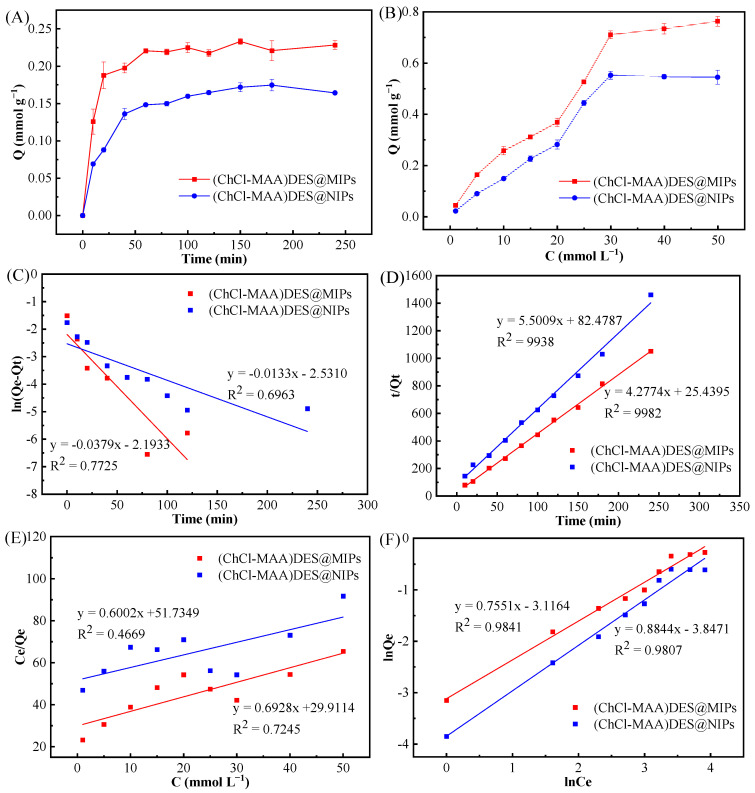
Adsorption kinetics curves (**A**), adsorption isotherms curves (**B**), pseudo-first-order kinetic model (**C**), pseudo-second-order kinetic model (**D**), Langmuir adsorption model (**E**), and Freundlich adsorption model (**F**) of GA on (ChCl-MAA)DES@MIPs or (ChCl-MAA)DES@NIPs.

The adsorption kinetics of (ChCl-MAA)DES@MIPs and (ChCl-MAA)DES@NIPs at 298 K were investigated to determine the adsorption rate and equilibrium time of GA. The dynamic adsorption curve of the adsorption capacity with the increase in adsorption time is shown in Figure 7A. It can be observed that the adsorption capacity of GA on (ChCl-MAA)DES@MIPs increased with increasing adsorption time and tended to reach equilibrium at about 60 min.

To further investigate the adsorption kinetics, the pseudo-first-order kinetic Equation (1) and pseudo-second-order kinetic Equation (2) were used to fit the dynamic adsorption data of (ChCl-MAA)DES@MIPs and (ChCl-MAA)DES@NIPs at 298 K, respectively.

ln(*Q*_e_ − *Q*_t_) = ln*Q*_e_ − k_1_*t*, (1)

*t*/*Q*_t_ = 1/(k_2_*Q*_e_) *+ t*/*Q*_e_, (2)

where *Q*_e_ and *Q*_t_ are the adsorption capacity at equilibrium and moment *t*; k_1_ and k_2_ are the rate constants of the two kinetics models, respectively. The corresponding parameters were calculated as Table 2.

After data fitting, pseudo-first-order and pseudo-second-order kinetic fitting diagrams of (ChCl-MAA)DES@MIPs (Figure 7C) and (ChCl-MAA)DES@NIPs (Figure 7D) were obtained. It was obvious that the correlation coefficients of pseudo-second-order kinetic fitting were larger than those of pseudo-first-order kinetic fitting, and the simulated adsorption quantities of *Q*_e_ were also closer to the actual adsorption quantity of *Q*_e_(exp). Therefore, adsorption of GA on the prepared MIPs would be conformed to the pseudo-second-order kinetic equation, which indicated the presence of external and internal diffusion of the adsorption at the same time. In a certain concentration range, the adsorption rate was positively correlated with the concentration of adsorbent as well as with the amount of adsorbent. When the amount of adsorbent is constant, the growth of adsorption rate was restricted due to the limited number of adsorption sites on the surface of the adsorbent, so there was a maximum value of the adsorption rate [43].

#### 2.3.2. Adsorption Thermodynamics

The adsorption isotherms curves of (ChCl-MAA)DES@MIPs and (ChCl-MAA)DES@NIPs changed with the increase in GA concentration at 298 K (Figure 7B). It can be noticed that the equilibrium adsorption capacity of MIPs was 1.3 times that of NIPs at the same temperature, which is probably due to the imprinted cavities of MIPs with GA.

Langmuir adsorption isotherm Equation (3) and Freundlich adsorption isotherm Equation (4) were fitted for (ChCl-MAA)DES@MIPs (NIPs) static adsorption data of the GA solution with different concentrations.

1/*Q*_e_ = 1/*Q*_m_ + 1/(*Q*_m_K_L_*c*_e_), (3)

ln*Q*_e_ = mln*c*_e_ + lnK_F_, (4)

where K_L_ is the Langmuir constant, and K_F_ is the Freundlich constant. In addition, m is the Freundlich characteristic adsorption parameter. *Q*_m_ and *Q*_e_ represent the maximum adsorption quantity and the adsorption quantity at *c*_e_, respectively. The fitting results are shown in Table 3.

The Langmuir equation represents the monolayer adsorption process, and the Freundlich equation introduces intermolecular force to represent the multilayer adsorption process. As presented in Table 3, the correlation coefficient of the Langmuir adsorption model (R^2^ = 0.7745) was lower than that of the Freundlich adsorption model (R^2^ = 0.9841). Therefore, the isothermal adsorption behavior of GA on DES@MIPs can be considered as multilayer adsorption.

Furthermore, a thorough review of the literature revealed a large number of studies on GA adsorption. Notably, the adsorption capacity of GA on MIPs prepared in this work considerably improved in comparison with those in the previous literature (Table 4).

#### 2.3.3. Adsorptive Selectivity and Reusability of DES@MIPs

**Figure 8:**
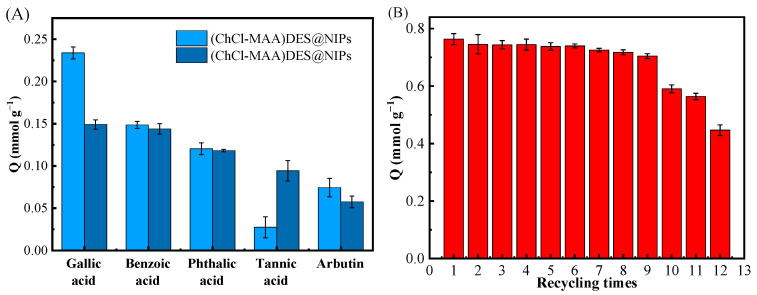
Competitive adsorption of GA, benzoic acid, phthalic acid, tannic acid, and arbutin on (ChCl-MAA)DES@MIPs or (ChCl-MAA)DES@NIPs at 298 K (**A**); the recycling times of adsorption/desorption of GA by (ChCl-MAA)DES@MIPs (**B**).

Figure 8A showed the competitive adsorption of GA, benzoic acid, phthalic acid, tannic acid, and arbutin on (ChCl-MAA)DES@MIPs at 298 K. It can be noticed that the selective adsorption capacity of GA on (ChCl-MAA)DES@MIPs was remarkably superior to the other four substances. Figure 8B shows the higher reusability of (ChCl-MAA)DES@MIPs, as the adsorption capacity of MMIPs remained above 90% after nine cycles. The imprinting factor α_GA_ (1.5667) was greater than α_other_ (Table 5), which indicated that (ChCl-MAA)DES@MIPs could be used to selectively separate GA from the mixture solution.

#### 2.3.4. Purification of GA from *Camellia* spp. Fruit Shells Using DES@MIPs

As shown in Table 6, the highest GA content of *Camellia polyodonta* was up to 5.81 mg kg^−1^, which was 7.8 times higher than that of *Camellia grijsii*. The results indicated that there was a great difference in GA content of *Camellia* spp. fruit shells among different varieties (58.15 mg kg^−1^ for *Camellia polyodonta*, 29.72 mg kg^−1^ for *Camellia oleifera* cultivar ‘Deyou 2’, 7.50 mg kg^−1^ for *Camellia oleifera* var. *monosperma*, 9.29 mg kg^−1^ for *Camellia pitardii*, and 7.43 mg kg^−1^ for *Camellia grijsii*, respectively). In addition, after GA in the real samples of different *Camellia* spp. fruit shells were adsorbed by (ChCl-MAA)DES@MIPs before eluted by methanol-acetic acid (9/1, *v*/*v*), the recovery of GA from *Camellia* spp. fruit shells was relatively high, ranging from 87.85–96.75%, and the purity of GA was above 80%. The results showed that (ChCl-MAA)DES@MIPs had better separation and purification ability of GA in the complex matrix and was universal for purification of gallic acid from different *Camellia* spp. fruit shells.

## 3. Materials and Methods

### 3.1. Chemicals and Apparatus

Ethylene glycol dimethacrylate (EGDMA, 98.0%), α-methylacrylic acid (MAA, 99.0%), and 2,2′-azobis-isobutyronitrile (AIBN, 98.0%) were supplied by Shanghai McLean Biochemical Technology Co., Ltd. (Shanghai, China). Absolute ethanol (≥99.7%), acetonitrile (≥99.8%), methanol (99.8%), choline chloride (ChCl, ≥98.0%), acyclic acid (AA, 99.0%), benzoic acid (99.5%), and dimethyl sulfoxide (DMSO, ≥99.5%) were purchased from Sinopharm Chemical Reagents Co., Ltd. (Beijing, China). Gallic acid (GA, 99.0%) was supplied by J&K Scientific Ltd. (Beijing, China). Phthalic acid (99.8%) was obtained from Tianjin Fuchen Chemical Reagent Co., Ltd. (Tianjin, China), tannic acid (99%) obtained from Tianjin Zhiyuan Chemical Reagent Co., Ltd. (Tianjin, China), and arbutin (98%) obtained from Shanghai Bide Pharmaceutical Technology Co., Ltd. (Shanghai, China). The solutions were prepared with deionized water without any further purification for any chemicals.

Scanning electron microscopy (SEM, TESCAN MIRA LMS, Czech Republic), particle size analysis (Mastersizer 2000, Malvern, London, UK), Fourier transform infrared spectroscopy (FT-IR, Thermo Nexus 870, Waltham, MA, USA), elemental analysis (EA, Elementar Vario el III, Frankfurt, Germany), nitrogen sorption porosimetry (Quadrasorb Si-3MP, Boynton Beach, FL, USA), and thermal gravimetry analysis (TGA, Tg-DTA 7300, Tokyo, Japan) were used to characterize the structure of MIPs and DES@MIPs. In detail, SEM, particle size analysis, and nitrogen sorption porosimetry were used to observe the appearance, particle size, and pore size distribution, respectively. FT-IR and EA were used for analyzing the structure of functional groups and the composition of chemical elementals, respectively. TGA was used to investigate thermal stability of DES@MIPs. HPLC analysis was carried out on the Purkinje L600 HPLC system (Beijing, China) with a UV-vis absorption detector.

### 3.2. Camellia spp. Fruit Shells and Sampling

Fruit shells of five different *Camellia* spp. including Camellia polyodonta, Camellia oleifera cultivar ‘Deyou 2’, Camellia oleifera var. monosperma, Camellia pitardii, and Camellia grijsii were collected from a different place of origin in October 2021 (Table 7). They were identified by Professor Deyi Yuan from Central South University of Forestry and Technology, Changsha, China, and voucher specimens were stored at the herbarium of College of Sciences, Central South University of Forestry and Technology, Changsha, China, under the No. CS211001-CS211005. After collection, the fruit shells were instantly dried at 40 °C in an oven with air circulation to a constant dry weight (DW) and then crushed to 98–110 μm in diameter. Each 10 g of the powder of *Camellia* spp. Fruit shells was taken, and the antioxidant components in the shells were completely extracted by percolation with 50% ethanol in water. The combined extract was concentrated to dry powder by rotary evaporation at 45 °C and kept in a refrigerator at 4 °C for further experiments. The varieties, origins, and yield of 50% ethanol extract for different *Camellia* spp. fruit shells were shown in Table 7.

### 3.3. Preparation of DES@MIPs

#### 3.3.1. Preparation of DES Functional Monomer

**Figure 9:**
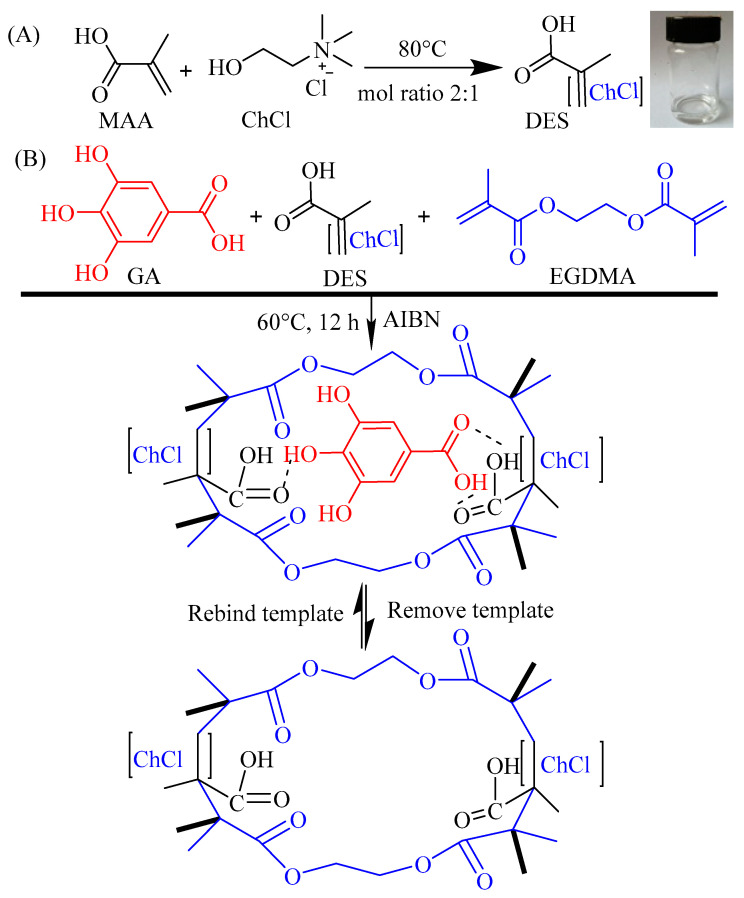
Schematic illustration of the procedure for preparing the DES with ChCl and MAA (**A**) and the DES@MIPswith DES as functional monomers (**B**).

The DES functional monomer was prepared in a typical thermochemical process [42,49] (Figure 9A): HBD and HBA were mixed in a molar ratio of 2:1 and heated at 80 °C until a transparent solution was obtained. MAA (2 mmol) or AA (2 mmol) was used for HBD, and ChCl (1 mmol) was used for HBA in this study.

#### 3.3.2. Synthesis of DES@MIPs

The process of preparing polymers using the bulk polymerization method [36,50] is simply described as the following (Figure 9B). Firstly, GA and DES functional monomer were dissolved in absolute ethanol with ultrasonic treatment for 10 min and stored at 4 °C for 8 h to prepare a preassembled solution. Then, cross-linker EGDMA and initiator AIBN were dissolved in the preassembled solution with ultrasonic treatment for 10 min, purged with N_2_ for 5 min, and reacted at 60 °C for 12 h. After polymerization, the DES@MIPs were ground into particles with diameters of 50 µm. Finally, the DES@MIPs were washed with methanol-acetic acid (9/1, *v*/*v*) under stirring reflux at 80 °C to remove GA, and then the DES@MIPs were washed with methanol to pH = 7 and dried at 50 °C. The same procedures were applied to prepare the molecularly non-imprinted polymers (DES@NIPs) as the control of DES@MIPs.

### 3.4. HPLC Analysis

HPLC analysis was performed on a Pgrandsil-STC-C_18_ column (4.6 mm × 150 mm, 5 µm) with 0.1% phosphoric acid in H_2_O and methanol (85:15, *v*/*v*) as mobile phase at a flow rate of 1.0 mL min^−1^, injection volume of 20 µL, column temperature of 30 °C, and detection wavelength of 260 nm.

### 3.5. Adsorption Experiment of DES@MIPs

The adsorption capacity (*Q*) was calculated using Equation (5) [51].

*Q*_t_ = (*c*_0_ − *c*_t_) *V/m*, (5)

where *c*_0_ and *c*_t_ (mmol L^−1^) are the initial and the equilibrium concentrations of the solution, respectively. *V* (L) is the volume of the solution, and *m* (g) is the mass of the DES@MIPs or DES@NIPs added.

#### 3.5.1. Kinetic Adsorption Experiment

For kinetic adsorption experiments, DES@MIP or DES@NIP (20.0 mg) was mixed with 1 mL GA aqueous solution (10.0 mmol L^−1^). The mixtures were shaken under 180 rpm at 308 K. The concentrations of GA were analyzed by HPLC at a certain interval (10, 20, 40, 60, 80, 100, 120, 150, 180, and 240 min), and the adsorption capacity *Q*_t_ (mmol g^−1^) was calculated by Equation (5).

#### 3.5.2. Isothermal Adsorption Experiment

For thermodynamic adsorption experiments, DES@MIPs or DES@NIPs (20.0 mg) was suspended in 1.0 mL GA aqueous solution at initial concentrations of 1.0 to 50.0 mmol L^−1^. The supernatant was analyzed by HPLC after shaking at 180 rpm under 298 K for 60 min. The equilibrium adsorption capacity *Q*_e_ (mmol g^−1^) of GA was calculated according to Equation (5), similarly to *Q*_t_.

#### 3.5.3. Selective Adsorption Experiment

DES@MIPs or DES@NIPs (20.0 mg) were suspended in mixed standard aqueous solutions (1.0 mL) of GA and four structural analogues (benzoic acid, phthalic acid, tannic acid, and arbutin) with the same initial concentration (10.0 mmol L^−1^) for selective adsorption. After shaking under 180 rpm at 298 K for 60 min, the supernatant was analyzed by HPLC, and the equilibrium adsorption capacity *Q*_e_ of the four standard substances was measured using Equation (5), similarly to *Q*_t_.

The specific recognition ability of the DES@MIPs or DES@NIPs could be assessed by imprinting factor (α), which was calculated using Equation (6).

α = *Q*_DES@MIPs_/*Q*_DES@NIPs_, (6)

where *Q*_DES@MIPs_ and *Q*_DES@NIPs_ mean the adsorption capacity of GA on DES@MIPs and DES@NIPs, respectively. The selectivity of recognition was assessed by the isolate factor (β), which was calculated using Equation (7).

β = α_GA_/α_other_, (7)

where α_GA_ is the imprinting factor for GA and α_other_ is the imprinting factor for other structural analogues.

#### 3.5.4. Regeneration and Reused Experiment

Regeneration and reused experiment was performed by adding 20 mg of (ChCl-MAA)DES@MIPs to 1 mL of 50 mmol L^−1^ GA and shaking at 180 rpm for 1 h at 298 K. After determining the GA equilibrium concentration *c*_t_ by HPLC, the adsorbed amount *Q*_t_ was calculated according to Equation (5). In each cycle, (ChCl-MAA)DES@MIPs adsorbed with GA were washed with methanol-acetic acid (9/1, *v*/*v*) by ultrasonication until the GA was completely removed cleanly as judged by HPLC. The above procedure was performed for 12 cycles of adsorption/desorption.

#### 3.5.5. GA Adsorption by DES@MIPs from *Camellia* spp. Fruit Shell Extract

Different masses of dry extract of *Camellia* spp. fruit shells were dissolved in DMSO before they were diluted with distilled water. (ChCl-MAA)DES@MIPs (100 mg) were given to 1 mL of the above diluted solution and shaken at 298 K for 60 min. The concentration changes of GA in the sample solution before and after adsorption was detected by HPLC.

## 4. Conclusions

In this work, (ChCl-MAA)DES@MIPs were prepared, characterized, and evaluated for their specific recognition of GA in *Camellia* spp. fruit shells. Compared with other MIPs using conventional functional monomers, DES@MIPs showed the superiority of favorable economic and environmental benefits after introducing the DES into the MIP framework. In addition, the resultant (ChCl-MAA)DES@MIPs are fissile mesoporous materials, with excellent thermal stability, adsorption capacity, selectivity, and reusability. The purification recovery of GA from different *Camellia* spp. fruit shells could range from 87.85–96.75% with a purity over 80%, which concluded that (ChCl-MAA)DES@MIPs are effective adsorbents and have universality for separation of target substances from complex systems. Given their large adsorption capacity, good selectivity, and universality, DES@MIPs could be further used to produce many other useful substances with bioactivity from *Camellia* spp. fruit shells or other agricultural wastes.

## Figures and Tables

**Table 1:** Data of pore size and specific surface area.

Polymers	Average Pore Diameter (nm)	Total Pore Volume (cm^3^ g^−1^)	Specific Surface Area (m^2^ g^−1^)
(ChCl-MAA)DES@MIPs	9.65	0.315	193.9
(ChCl-MAA)DES@NIPs	11.64	0.373	189.6

**Table 2:** Simulation parameters of pseudo-first-order and pseudo-second-order kinetic equations at 298 K.

Polymers	Q_e_(exp) (mmol g^−1^)	Pseudo-First-Order Kinetic Model			Pseudo-Second-Order Kinetic Model		
		Q_e_ (mmol g^−1^)	k_1_ (min^−1^)	R^2^	Q_e_ (mmol g^−1^)	k_2_ (g mmol^−1^ min^−1^)	R^2^
DES@MIPs	0.2206 ± 0.0030	0.1115	0.0379	0.7725	0.2338	0.1705	0.9982
DES@NIPs	0.1717 ± 0.0059	0.0796	0.0133	0.0696	0.1818	0.0666	0.9938

**Table 3:** Parameters of Langmuir equation and Freundlich equation.

Polymers	Q_e_(exp) (mmol g^−1^)	Langmuir Isotherm Model			Freundlich Isotherm Model		
		Q_m_ (mmol g^−1^)	k_L_ (min^−1^)	R_1_^2^	K_F_ (mmol g^−1^)	m (g mmol^−1^ min^−1^)	R_2_^2^
DES@MIPs	0.7110 ± 0.0152	1.4434	0.0483	0.7745	0.0443	0.7551	0.9841
DES@NIPs	0.5524 ± 0.0153	1.6661	0.0322	0.4669	0.0213	0.8844	0.9807

**Table 4:** Comparison adsorption capacity of GA-enriched MIPs reported in the literature.

Monomers	Adsorption Capacity (mmol g^−1^)	Reference
Dopamine	0.5214	[2]
Dopamine	0.5978	[44]
Acrylamide	0.1458	[45]
Acrylic acid	0.3468	[46]
Acrylonitrile	0.4585	[46]
2-hydroxyethyl methacrylate	0.3997	[46]
Resorcinol and melamine	0.2839	[47]
4-vinylpyridine	0.2086	[48]
4-vinylpyridine	0.2444	[29]
(ChCl-MAA)DES	0.7110	This work

**Table 5:** Imprinting factor α and isolate factors β of DES@MIPs with GA as template.

Factors	Gallic Acid	Benzoic Acid	Phthalic Acid	Tannic Acid	Arbutin
α	1.5667	1.0323	1.0187	0.4068	1.1888
β	/	1.5177	1.5379	3.8513	1.3179

**Table 6:** Effects of DES@MIPs on the recovery of GA from different *Camellia* spp. fruit shells.

Varieties of *Camellia* spp.	Content of GA in Fruit Shells (mg kg^−1^)	Recovery (%)	Purity of GA (%)
*Camellia polyodonta*	58.15 ± 2.07	96.75 ± 3.07	97.45 ± 2.10
*Camellia oleifera* cultivar ‘Deyou 2’	29.72 ± 1.15	93.80 ± 4.23	90.52 ± 5.54
*Camellia oleifera var. monosperma*	7.50 ± 0.57	87.85 ± 3.21	80.64 ± 3.40
*Camellia pitardii*	9.29 ± 0.46	88.78 ± 2.76	82.16 ± 3.92
*Camellia grijsii*	7.43 ± 0.24	88.05 ± 4.13	82.29 ± 2.98

**Table 7:** The varieties, origins, and yield of 50% ethanol extract for different *Camellia* spp. fruit shells.

Varieties	Origins	Yield (%)
*Camellia polyodonta*	Changsha, Hunan	16.49
*Camellia oleifera* cultivar ‘Deyou 2’	Changsha, Hunan	13.02
*Camellia oleifera var. monosperma*	Liping, Guizhou	11.42
*Camellia pitardii*	Liping, Guizhou	23.36
*Camellia grijsii*	Liping, Guizhou	16.56

## Data Availability

All data relevant to the study are included within the article.
